# Do reverse total shoulder replacements have better clinical and functional outcomes than hemiarthroplasty for patients undergoing proximal humeral tumor resection using devitalized autograft composite reconstruction: a case-control study

**DOI:** 10.1186/s13018-021-02488-1

**Published:** 2021-07-14

**Authors:** Dongqing Zuo, Haoran Mu, Qingbo Yang, Mengxiong Sun, Jiakang Shen, Hongsheng Wang, Xiaojun Ma, Chongren Wang, Chuanping Li, Wei Sun, Zhengdong Cai

**Affiliations:** 1 Department of Orthopedic Oncology, Shanghai General Hospital Affiliated with Shanghai Jiaotong University, No. 100 Haining Road, Hongkou District, Shanghai, 200080 China; 2 Shanghai Bone Tumor Institute, Shanghai, China; 3 Department of Thoracic Surgery, Shanghai Tenth People’s Hospital affiliated with Tongji University, 301 Yanchang Road, Shanghai, China; 4 Department of Rehabilitation, Shanghai General Hospital Affiliated with Shanghai Jiaotong University, No. 100 Haining Road, Shanghai, China

**Keywords:** Reverse total shoulder arthroplasty, Hemiarthroplasty, Devitalized autograft

## Abstract

**Objective:**

To compare the efficacy and prognosis of reverse total shoulder arthroplasty (rTSA) with shoulder hemiarthroplasty (SHA) using devitalized autograft or allograft composite reconstruction after proximal humeral tumor resection.

**Methods:**

We retrospectively reviewed patients who underwent SHA (32) and rTSA (20) for tumor resections of the proximal humerus from January 2014 to July 2020. The clinical results included duration of the operation, intraoperative blood loss, bone union, visual analog scale (VAS) score, shoulder range of motion (ROM), American Shoulder and Elbow Surgeons (ASES) shoulder score, recurrence, and overall survival.

**Results:**

Fifty-two patients were followed up for a mean of 30 months. Thirty-two patients were SHA with allograft-prosthetic composite (APC) reconstructions, while other 20 were rTSA with devitalized autograft-prosthetic composite reconstructions. At the end of the follow-up, 2 recurrence, 3 postoperative infections, and 4 subluxations occurred among the SHA patients. Two patients in the rTSA group had postoperative anterior dislocation and underwent revision surgery with surgical mesh, and 2 (2/20) had grade II scapular notching. The mean VAS score of the shoulder was 1.5 ± 0.8 in the rTSA group and 2.3 ± 1.2 in the SHA group (p < 0.05). The mean active forward flexion of the shoulder joint was 50.6 ± 6.0 in the SHA group and 100 ± 7.6 in the rTSA group (p < 0.05). The ASES shoulder score was 78 ± 3.0 in the rTSA group and 52 ± 5.6 in the SHA group (p < 0.05). The overall 3-year survival rate of all patients was 60.0%, and patients in the rTSA group showed better survival in terms of the mean 3-year OS than patients in the SHA group (p = 0.04).

**Conclusion:**

rTSA with devitalized autograft-prosthetic composite can offer a reasonable reconstruction of the shoulder joint after Malawer type I tumor resection. Compared with patients who underwent SHA, patients who underwent rTSA present good outcomes, a better range of motion, better bone union, and no increase in instability rate in the mid-term.

## Background

The proximal humerus is a common location for both primary and metastatic bone tumors, the second most common site for all osseous sarcomas and the third most common site for osteosarcoma [1, 2]. The main goals of shoulder reconstruction are to restore function and limit complications. Many reconstruction and stabilization options are available after principle surgical management, including allografts, allograft-prosthetic composites (APCs), mega-prostheses, and more recently, reverse total shoulder arthroplasty (rTSA). Patient activity, tumor characteristics, and anatomic involvement are important factors to consider when selecting the optimal reconstruction. The function of the affected limb can be acceptable even if shoulder elevation is restricted. Fuhrmann et al. [3] reported that if the remaining muscle is not sufficient to centralize the joint, the effect of a prosthesis would not differ from that of a spacer. However, there is no consensus regarding the best reconstructive technique following proximal humerus resection. Limb salvage therapy for tumors of the shoulder is extremely challenging because the shoulder girdle is essentially an unstable anatomical structure requiring complex static (scapula, clavicle, humerus, joint capsule, ligaments, etc.) and dynamic stabilization systems (deltoid muscles, rotator cuffs) to maintain performance, which are affected following extensive surgical resection during shoulder tumor resection [4]. Malawer et al. [5] proposed a shoulder girdle surgical classification according to the extent of the surgical resection area, in which shoulder girdle tumor surgery is divided into 6 types: intraarticular proximal humerus resection (type I), extra-articular proximal humerus resection (type II), intraarticular total humerus resection (type III), extra-articular scapular and humeral head resection (type IV), extra-articular total humerus, and glenoid resection (type V), and extra-articular humerus and scapular resection (VI). It is worth noting that the type of resection does not depend on the reconstruction method and that extra-articular resection (IV ~ VI type) is important for cases where tumors may involve the shoulder joints.

rTSA devices were designed in the early 1970s and were mainly used in the treatment of severe osteoarthritis or rheumatoid arthritis with rotator cuff defects at first, although recently, they are generally accepted [6]. Their special biomechanical structure provides different mechanisms for shoulder joint movement; that is, it relies not on the integrity of the rotator cuff but rather on the strength of the deltoid muscle to produce good abduction, forward flexion and lifting ability in the shoulder joint. Studies have shown that rTSA is a feasible and effective method for reconstructing bone defects after tumor resection unless the patient has axillary nerve injury and a nonfunctional deltoid muscle [7–9]. However, some problems remain with the underlying operation, such as complications, scapular notching, the repair of limb bone defects after large-scale tumor resection, and soft-tissue reconstruction after deltoid muscle resection [10].

APC reconstruction has been used at various anatomic sites to improve mechanical support and the union of biological reconstruction composites. Few studies have compared the efficacy and functional results of rTSA and shoulder hemiarthroplasty (SHA) with APC or devitalized autograft-prosthetic composites. The current study aims to evaluate the efficacy and mid-term functional results of SHA versus rTSA after Malawer type I proximal humeral tumor resection with both devitalized autografts for bone defects in rTSA reconstruction.

## Materials and methods

### Study cohort

We retrospectively reviewed patients aged between 18 and 75 years who underwent proximal humeral reconstruction with shoulder SHA or rTSA for malignant or progressively bone or soft-tissue tumors at Shanghai General Hospital of Shanghai Jiaotong University (Shanghai) between April 2014 and July 2020. Forty-seven patients were included in our study. All patients were classified as type I in the Malawer surgical classification system for shoulder girdle resection [5]. Tumor staging was determined according to the Musculoskeletal Tumor Society (MSTS) Staging System [11] using local radiography, computed tomography (CT) of the chest, and magnetic resonance imaging (MRI). The demographic and clinical data of the patients are detailed in Table 1.

**Table 1:** Patient demographic characteristics

Parameters	rTSA N=20	SHA N=32	*P* value
Sex (M/F)	8/12	18/14	0.2629
Age, mean ± SD	46.2 ± 2.5	56 ± 3.2	< 0.0001
Primary bone malignancy	15/20	8/32	0.0002
Metastatic tumors	3/20	18/32	0.0026
Aggressive bone tumors	2/20	6/32	0.4048
Operative duration	145 ± 24	120 ± 40	0.0150
Length of bone resection	8.5 ± 3.8	12 ± 4.8	0.0080
Allograft bone	0/20	28/32	-
Devitalized bone	20/20	0/32	-
Follow-up duration	33.8 ± 3.2	29.7 ± 4.5	0.0009
Surgical mesh	15/20	20/32	0.3579
Blood loss	800 ± 35	650 ± 60	< 0.0001

### Preoperative management and assessment

All patients underwent preoperative radiologic evaluation, including X-rays, CT scans, and nuclear MRI scans, to evaluate the tumor margin and the degrees of bone destruction and soft-tissue involvement. Pathology was confirmed by bone biopsy under digital radiography guidance before definitive tumor resection. For primary and mono-metastatic lesions, positron emission tomography-computed tomography (PET-CT) was performed. No patients were confirmed to have axillary neurovascular bundle involvement.

### Tumor resection and devitalized autograft-prosthetic composite rTSA reconstructions

In brief, general anesthesia combined with a brachial plexus block was applied to all patients. The shoulder and humeral diaphyses were exposed through a deltopectoral approach in a beach chair position. The extended deltopectoral interval approach was approximately 10-15 cm long. The osteotomy was evaluated with preoperative MRI. Generally, humeral osteotomy was performed with a wire saw 2-3 cm from the distal end of the tumor. The tendon of the rotator cuff was resected 1-1.5 cm from the greater and lesser tuberosity. The resected ends of the infraspinatus, teres minor, and subscapular muscles were marked with sutures. The axillary nerve was exposed and protected, and the joint capsule was thoroughly loosened. For the rTSA procedure, the glenoid fossa was exposed, the glenoid component was implanted in a satisfactory position, and after adequate contact with healthy native bone was ensured, secure component fixation was obtained. When a malignant tumor was resected, the length of the affected limb was matched to the length of the resection as measured intraoperatively. In certain circumstances, the autograft could be resected a few millimeters longer than the anticipated required length to allow fine adjustments and obtain optimal fits and osteosynthesis junction. After resection of tumor segment, the autograft is prepared on the back table, the tumor tissue was removed carefully from the segment, and then autograft usually is devitalized by high-speed burring and dehydrated alcohol for 30 min, preparing the medullary reaming to receive the humeral stem. Prior insertion of humeral stem, fills the devitalized proximal humeral segment with bone cement, inserts the humeral stem and carefully removes the bone cement from the osteotomy.

The attached autograft tendon was preserved, and the tendon-bone interface was restored by drilling the autograft bone, if needed. After the distal humerus was reamed, a pulp plug was placed, and bone cement was inserted into the prosthesis of the humeral stalk. Once the cement was completely cured, trial implants were used to select the ideal thickness of the humeral bearing. The ideal soft-tissue tension was judged by the ease of relocation and the tension on the deltoid, conjoined tendon, and other structures, and the range of motion should be free of impingement. The humeral polyethylene bearing was impacted onto the upper portion of the humeral component, and the joint was relocated. Soft-tissue reconstruction depended on the remaining tissue after performing adequate resection for the oncologic portion of the procedure. If needed, the tendons of the posterosuperior cuff, subscapularis, deltoid, and/or pectoralis major were attached to their autograft counterparts in an attempt to provide stability and potentially a better line of pull for internal/external rotation in abduction. For most rTSA patient, surgical mesh were applied, the insertion of cuff was marked with tendon suture carefully after resection of tumor. The surgical mesh was cut to proper size to pack the humeral stem after cemented prosthesis were fixed, the tendon suture of rotator cuff and remaining joint capsula were sutured to the surgical mesh using the accessary insertion of the prosthesis.

The wound was closed in layers in a standard fashion. For hemiarthroplasty procedures, there were no obvious differences in tumor resection, osteotomy, soft-tissue repair, and wound closure.

### Postoperative management

Closed suction drainage was maintained for 24 to 48 h after surgery. Intravenous antibiotics, for example, cefuroxime commonly, were routinely used postoperatively to prevent infection for 3 days. Postoperative immobilization was used for all patients with a 45° abduction brace for 4 weeks; active functional exercise of the hand, wrist, and elbow joints was started the next day after the operation. After 1 month, the brace was removed, and patients started active mobilization with pendulum movements limited to 30° of abduction, forward flexion and extension, with a rehabilitation specialist helping prevent forced passive-assisted motion for the first weeks, followed by full range of motion (ROM) exercises.

### Follow-up and function assessment

The patients in the two groups were followed up at the 2nd week, the 6th week, the 3rd month, the 6th month, the 1st year, and every year up to 5 years after surgery. General follow-up assessments included physical examinations, plain radiographs, CT scans, measurements of the shoulder range of motion, and shoulder function. Functional evaluation of the shoulder joint was conducted using the American Shoulder and Elbow Surgeons (ASES) shoulder score. The presence and location of scapular notching in rTSA were determined on anteroposterior radiographs and 3D CT (coronal and axial sections) by consensus interpretation by the authors. Grading of the radiographs was performed using the system described by Sirveaux et al. [12]. Outcomes including recurrence, complications, and metastases were reported along with the 3-year overall survival (OS).

### Statistical analysis

All data were statistically analyzed using SPSS v.20.0 (SPSS, Inc., Chicago, IL, USA). Numerical data are expressed as the mean values ± SD, and categorical data are described as absolute frequencies. The 3-year OS rate and local recurrence between the two procedure groups were evaluated using chi-square tests or Fisher’s exact test. The ASES shoulder score was compared using Student’s t tests. All survival data, including OS, were analyzed using the Kaplan-Meier method and log-rank test. Statistical significance was defined as a P value less than or equal to 0.05 (P ≤ 0.05).

## Results

### Patient characteristics in the study cohort and follow-up results

Fifty-two patients were included in the current study, including 20 rTSA and 32 SHA patients (Table 1). In the rTSA group, there were 8 males and 12 females, and in the SHA group, there were 18 males and 14 males. The age of the patients in the rTSA group was 46.2 ± 2.5 years, and that in the SHA group was 56 ± 3.2 years (*p* < 0.05). Overall, the most frequent histopathologic tumor subtypes were chondrosarcoma (12) and giant cell tumor of bone (8), followed by 11 osteosarcomas, and 21 metastatic lesions, including 8 lung cancers, 6 renal cell carcinomas, 4 gastric cancers, 2 colorectal cancers, and 1 myeloma. The operative duration of the rTSA group was 145 ± 24 min, and that of the SHA group was 120 ± 40 min (P = 0.01). The length of the bone osteotomy for patients in the rTSA group was 8.5 ± 3.8 cm, and that for patients in the SHA group was 12 ± 4.8 cm (P = 0.008).

**Fig. 1:**
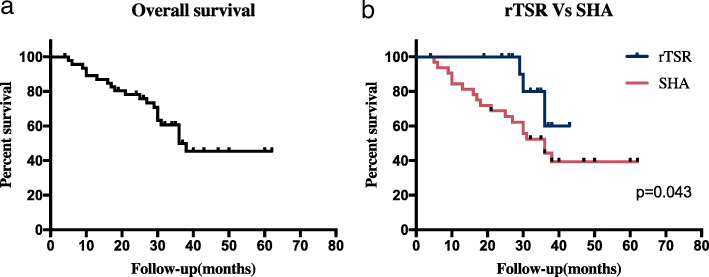
(**a**) The overall survival of patients during the follow-up; (**b**) comparison of the survival between rTSA patients and SHA patients during the follow-up

Fifty-two patients were followed up for a mean of approximately 30 months (3-60 months). At the end of the follow-up, 2 patients in the rTSA group had postoperative anterior dislocation and underwent revision surgery with surgical mesh, while 2 patients had grade II scapular notching in the rTSA group. And in the SHA group, 2 recurrence, 3 postoperative infections and 4 subluxations had occurred. The scores remained satisfactory, although late complications occurred. Three patients in the SHA group had superficial postoperative infections, 2 experienced wound healing after changing the dressings for 1 month, and one patient recovered after skin grafting for skin necrosis. One patient with chondrosarcoma of the proximal humerus in the SHA group had shoulder subluxation 1 month after surgery. Because of patient noncompliance with immobilization, the patient was then placed into a shoulder abduction brace for 1 month and performed exercises at a rehabilitation clinic with favorable functional results. Two patients in the SHA group had asymptomatic subluxation during the follow-up visit, and no further treatment was taken. Two patients in the rTSA group had postoperative anterior dislocations and underwent revision surgery with a surgical mesh. At the time of the last follow-up visit, no recurrence was observed in any surviving patients. No subsequent metastasis was confirmed in patients with a primary malignant tumor. The overall 3-year survival of all patients was 60.0%, and patients in the rTSA group showed better survival than patients in the SHA group (P = 0.043) (Fig. 1).

### Functional follow-up results of patients in the rTSA and SHA groups

Patients with rTSA reconstruction presented with better shoulder ROM in forward flexion and abduction than those who underwent SHA at the 12-month follow-up (Table 2). Two patients in the rTSA group had grade II scapular notching. The functional follow-up results demonstrated that the patients treated with rTSA achieved a better outcome. Significantly better bone union was seen in rTSA (16/20) than patients in SHA group (8/32) (p < 0.05). The mean visual analog scale (VAS) score of the shoulder was 1.5 ± 0.8 in the rTSA group and 2.3 ± 1.2 in the SHA group (P = 0.0112). The mean active forward flexion of the shoulder joint was 95 ± 5.6 in the rTSA group and 55.6 ± 8.0 in the SHA group (P = 0.0026). The ASES shoulder score was 78 ± 3.5 in the rTSA group and 52 ± 5.6 in the SHA group (P = 0.0040). We also observed a trend showing that patients in the SHA group demonstrated deteriorating functional scores at the 24-month and 36-month follow-up visits, while patients who underwent rTSA tended to have satisfactory and stable functional scores during follow-up.

**Table 2:** Clinical and functional follow-up outcomes

Parameters	rTSA N=20	Hemiarthroplasty N=32	*P* value
Revision surgeries	2/20	0/32	0.0704
Infections	0/20	3/32	0.1646
Local recurrence	0/20	2/32	0.2629
Dislocations	2/20	0/32	0.0704
Subluxations	0/20	4/32	0.1037
Bone unions (12 months)	16/20	8/32	< 0.0001
Bone resorptions (12 months)	4/20	24/32	< 0.0001
VAS score	1.5 ± 0.8	2.3 ± 1.2	0.0112
Forward flexion	95 ± 5.6	55.6 ± 8.0	< 0.0001
Abduction (at the end of follow-up)	110 ± 10	25.5 ± 5.0	< 0.0001
External rotation	25 ± 4.5	5 ± 1.5	< 0.0001
Abduction (12 months)	100 ± 7.6	50.6 ± 6.0	< 0.0001
ASES score, mean ± SD (12 months)	78 ± 3.5	52 ± 5.6	< 0.0001

### Typical rTSA and SHA cases in our study cohort.

#### Case 1: A patient who underwent SHA and APC reconstruction

**Fig. 2:**
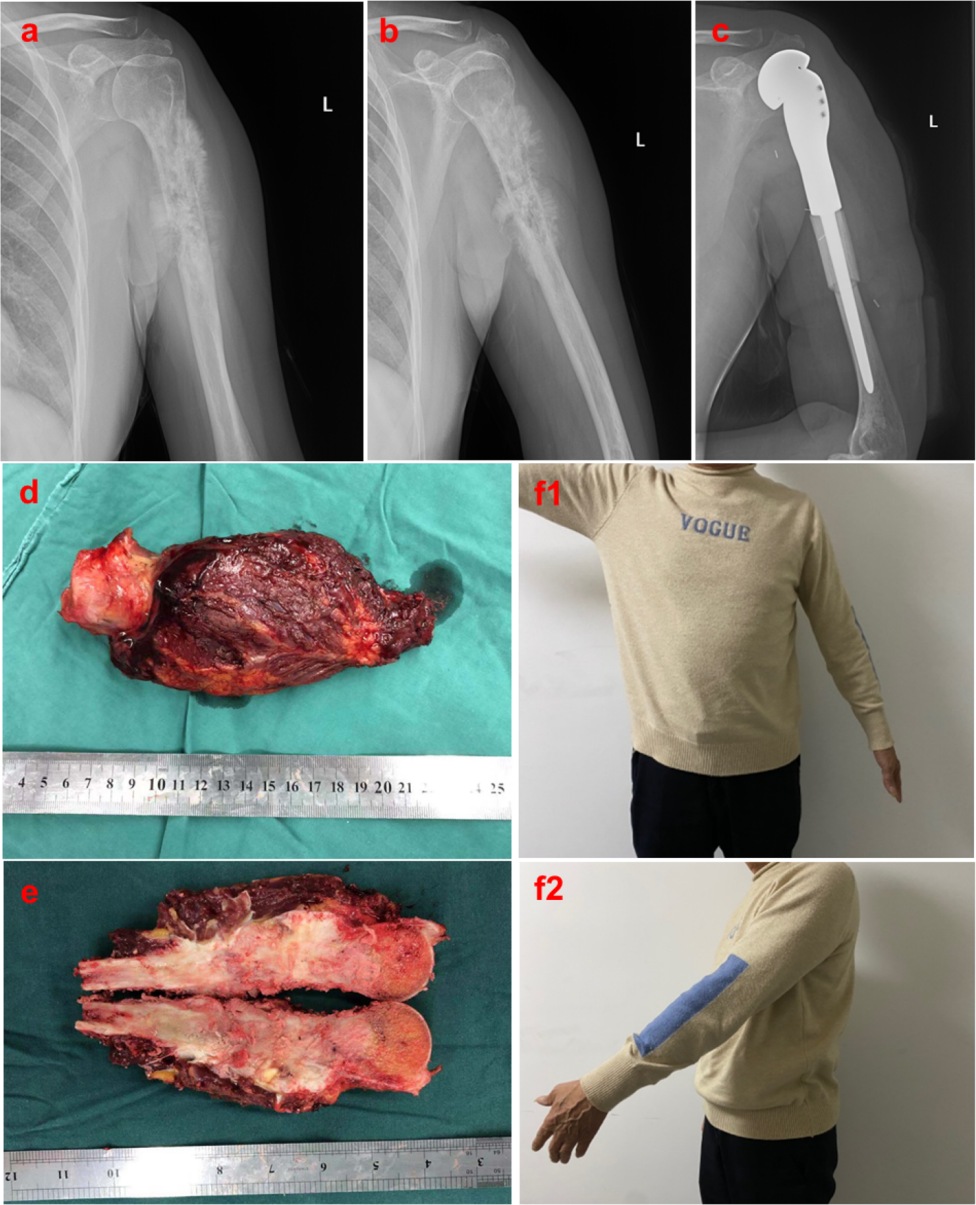
Case 1 (**a**, **b**) Preoperative AP X-ray indicates an osteogenic lesion and pathological fracture in the proximal humerus. (**c**) Six-month postoperative AP X-ray indicates stable joint function and alignment. (**d**, **e**) Surgical sample of the humeral osteosarcoma. (**f**1-2) Functional follow-up indicated stable joint function and alignment

A 55-year-old male who was diagnosed with humeral osteosarcoma (Enneking III) underwent SHA and APC reconstruction (Fig. 2). Preoperative anteroposterior (AP) X-ray indicated osteogenic lesions and pathological fractures in the proximal humerus. Postoperative AP X-ray 6 months later indicated stable joint function and alignment.

#### Case 2: A patient who underwent rTSA and autograft-prosthetic composite reconstruction

**Fig. 3:**
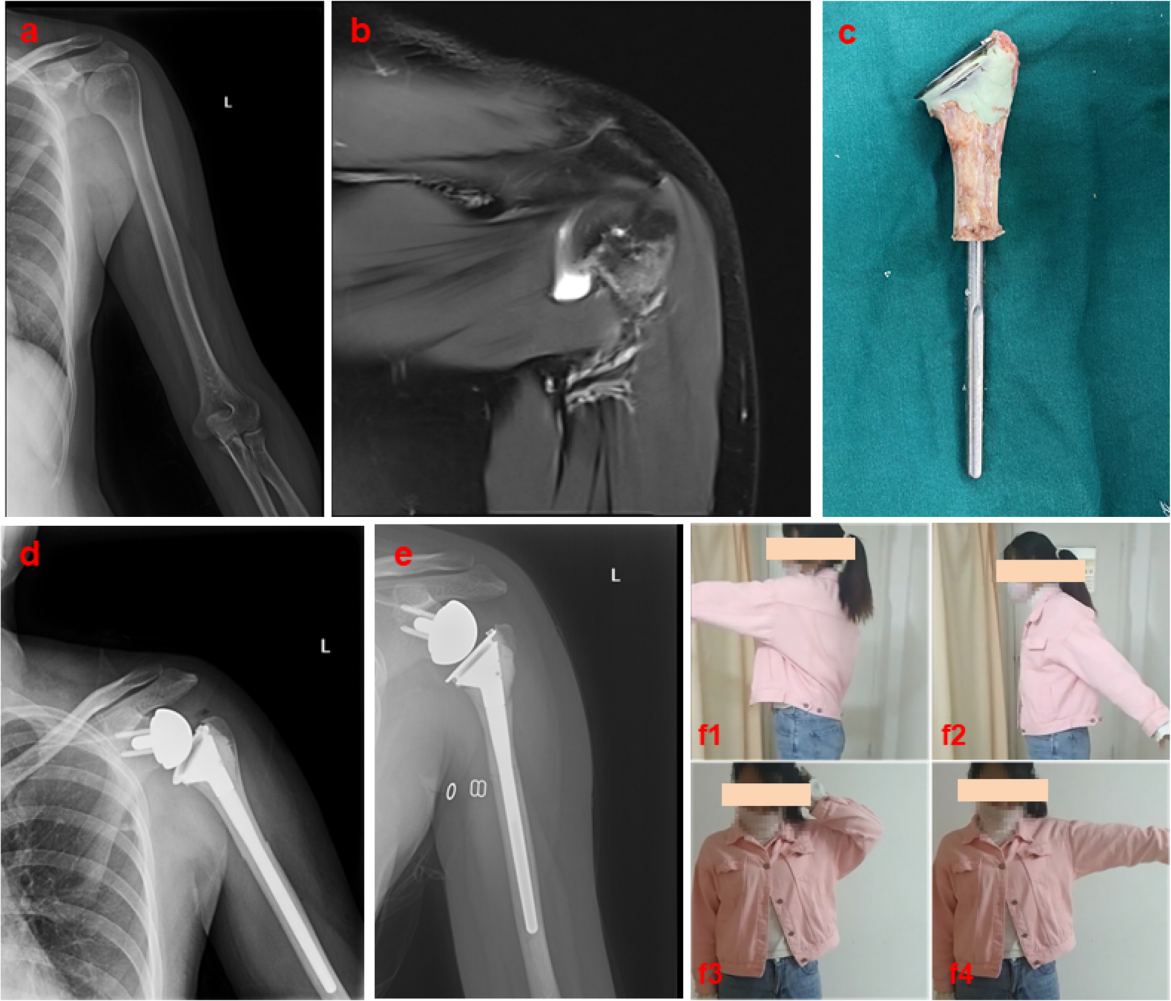
Case 2 (**a**, **b**) Preoperative AP X-ray and MRI indicate an osteogenic lesion and the pathological fracture in the left caput humeri. (**c**) Surgically inactivated isolated autogenous graft. (**d**) Postoperative AP X-ray. (**e**, **f**1-4) Six months later, the postoperative X-ray image and functional follow-up indicated stable joint function and alignment

A 31-year-old female who was diagnosed with left humeral osteosarcoma (Enneking IIB) underwent rTSA and devitalized autograft-prosthetic composite reconstruction (Fig. 3). Preoperative AP X-ray and MRI indicated an osteogenic lesion and pathological fracture in the left caput humeri. Six months later, postoperative X-ray images and functional follow-up indicated stable joint function and alignment.

## Discussion

Anatomic reconstruction of the proximal humerus performed for tumor resection involving rotator cuff insertions has been used to maintain a cosmetic appearance and shoulder stability, but the resulting shoulder function is typically being limited. Reconstruction options, including prostheses, biological reconstruction, and shoulder arthrodesis, have been reported with comparable oncological follow-up results in terms of the mean survival and tumor recurrence [1, 13–15]. However, several complications can result from the prosthesis procedures, including the risk of deep infection, loosening, proximal migration, distal subluxation, and periprosthetic fractures. Biological reconstructions such as arthrodesis [16–18], vascularized fibular grafts, clavicle pro humerus [19], and osteoarticular allografts have been reported to have a high incidence of complications, including bone graft failure, aseptic proximal pseudarthrosis, and necrosis of the skin [20]. Several case series have reported complication rates of approximately 20-50% for rTSA patients, including shoulder instability, dislocation, and aseptic loosening [7–9]. Several studies have reported encouraging case series results for rTSA in oncological reconstruction of the humerus, but only T.W. Grosel et al. [21] has directly compared the outcome and function details with rTSA and SHA reconstruction. In our current study, patient outcome and functional follow-up were assessed in rTSA group and SHA group, significant better function and bone union rate were reported in rTSA group, meanwhile the rate of complications or revision surgery was similar between hemiarthroplasty patients (8/32) and patients in the rTSA group (2/20). And no recurrence was indicated in rTSA group.

In the current study, patients with rTSA showed significantly better ROM in forward flexion and abduction. We reported a mean 100° active forward flexion among rTSA patients versus 50.6° among SHA patients. T.W. Grosel et al. [21] reported a mean forward flexion of 85° for rTSA patients and 28° for hemiarthroplasty patients. Lazerges et al. [22] reported similar functional results in a series of 6 rTSA patients, with a mean shoulder range of motion of 95 ± 5.6° for forward flexion. In a case series involving 8 patients, Maclean et al. [23] found a mean forward flexion of 71°. Our functional results for rTSA are similar to what has been reported, and we found a better shoulder function score and forward flexion and abduction range of motion than for APC hemiarthroplasty reconstruction. T.W. Grosel et al. [21] also compared rTSA with hemiarthroplasty and found that compared with hemiarthroplasty patients, rTSA patients had similar outcomes, a better range of motion, and no increase in the instability rate. This is in accordance with our research findings; rTSA patients in our study presented better functional scores and ranges of motion. In addition, rTSA patients have been reported to have an advantage in postoperative pain. Notably, the average length of the humerus osteotomies in the SHA group was longer than that in the rTSA group, which in turn may result in more soft-tissue release during tumor resection. We also observed that 4/20 rTSA and 24/32 SHA reconstruction patients presented with nonunion in 12 month follow-up visit. The higher VAS score among the SHA patients could be caused by the instability of APC reconstruction, and long-term follow-up or alternatives with modular prostheses may be a solution instead of APC reconstruction. Giulia Trovarelli et al. [24] investigated 22 patients who underwent reconstruction with modular rTSA. They reported one shoulder dislocation and one aseptic loosening out of four patients. The function in these patients on the outcome scales was generally satisfactory, and the implant survival in patients with major complications (aseptic loosening, breakage, and infection) was 94% 3 and 5 years postoperatively, showing obvious superiority in short-term function and implant complications to APC reconstruction. Scapular notching is frequently observed following rTSA. Joel Kolmodin et al. [25] reported that 59% (17/29) of patients had scapular notching after rTSA for rotator cuff tears. We observed that 2 of 20 rTSA patients had grade II notching. The relatively small number of patients and the non-randomized research design could also contribute to the current conclusion, and future studies should be performed using a larger sample size and a longer-term follow-up period.

In the current study, rTSA with devitalized autograft-prosthetic composite reconstruction showed significantly better function and bone union results than SHA with conventional APC. Allograft bone often serves as a mechanical support junction and spacer after bone segment resection in bone tumor surgery. Postoperative nonunion and graft bone resorption are very common during follow-up, while devitalized autografts provide anatomic support and precise reconstruction during bone union, thus reducing the possibility of bone loss and instability. Surprisingly, bone-graft nonunion only occurred in 4 patients (20%) who underwent pasteurized autograft-prosthetic composite reconstruction at the 12-month follow-up, which is relatively common among APC patients. Although the autograft is devitalized by high-speed burring and dehydrated alcohol, careful removal of the cement and close contact of the host bone and the devitalized autograft at the osteotomy location may contribute to bone union after surgery. Thus, we prefer devitalized autograft-prosthetic composite to traditional autograft-prosthetic composite reconstruction in patients with low-grade bone malignancy, especially in patients with localized lesions in the bone.

This study, to our knowledge, included more patients than any other published research, and the using of deviatalized bone combined with prosthesis in rTSA shoulder reconstruction is also a novelty. While it still had its limitation. Our study is a retrospective, non-randomized case-control study involving patients with heterogeneous diagnoses. The selection biases could be attributed to the fact that rTSA implants cost twice as much as conventional proximal humeral prostheses in China, and younger patients and those with a better prognosis were prioritized for rTSA reconstruction. This may have led to a better overall survival rate in the rTSA group than in the SHA group. Studies involved a larger cohort or a longer following-up will verify our results.

## Data Availability

Not applicable.

## Abbreviations

rTSA: Reverse total shoulder arthroplasty
SHA: Shoulder hemiarthroplasty
VAS: Visual analog scale
ROM: Shoulder range of motion
ASES: American Shoulder and Elbow Surgeons
APC: Allograft-prosthetic composite
MSTS: Musculoskeletal Tumor Society
CT: Computed tomography
MRI: Magnetic resonance imaging
PET-CT: Positron emission tomography-computed tomography
ROM: Range of motion
OS: Overall survival
